# High-resolution crop yield and water productivity dataset generated using random forest and remote sensing

**DOI:** 10.1038/s41597-022-01761-0

**Published:** 2022-10-21

**Authors:** Minghan Cheng, Xiyun Jiao, Lei Shi, Josep Penuelas, Lalit Kumar, Chenwei Nie, Tianao Wu, Kaihua Liu, Wenbin Wu, Xiuliang Jin

**Affiliations:** 1 Jiangsu Key Laboratory of Crop Genetics and Physiology/Jiangsu Key Laboratory of Crop Cultivation and Physiology, Agricultural College, Yangzhou University, 225009 Yangzhou, P.R. China; 2 Jiangsu Co-Innovation Center for Modern Production Technology of Grain Crops, Yangzhou University, 225009 Yangzhou, P.R. China; 3 Institute of Crop Sciences, Chinese Academy of Agricultural Sciences/Key Laboratory of Crop Physiology and Ecology, Ministry of Agriculture, Beijing, 100081 P.R. China; 4 College of Agricultural Science and Engineering, Hohai University, Nanjing, Jiangsu Province 210098 P.R. China; 5 CSIC, Global Ecology Unit CREAF-CSIC-UAB, Bellaterra, 08193 Barcelona, Catalonia Spain; 6 CREAF, Cerdanyola del Vallès, 08193 Barcelona, Catalonia Spain; 7 EastCoast Geospatial Consultants, Armidale, NSW 2350 Australia; 8 College of Hydrology and Water Resources, Hohai University, Nanjing, Jiangsu Province 210098 P.R. China; 9 Institute of Agricultural Resources and Regional Planning, Chinese Academy of Agricultural Sciences, 100081 Beijing, P.R. China; 10 National Nanfan Research Institute (Sanya), Chinese Academy of Agricultural Sciences, 572024 Sanya, China

**Keywords:** Hydrology, Agroecology

## Abstract

Accurate and high-resolution crop yield and crop water productivity (CWP) datasets are required to understand and predict spatiotemporal variation in agricultural production capacity; however, datasets for maize and wheat, two key staple dryland crops in China, are currently lacking. In this study, we generated and evaluated a long-term data series, at 1-km resolution of crop yield and CWP for maize and wheat across China, based on the multiple remotely sensed indicators and random forest algorithm. Results showed that MOD16 products are an accurate alternative to eddy covariance flux tower data to describe crop evapotranspiration (maize and wheat RMSE: 4.42 and 3.81 mm/8d, respectively) and the proposed yield estimation model showed accuracy at local (maize and wheat rRMSE: 26.81 and 21.80%, respectively) and regional (maize and wheat rRMSE: 15.36 and 17.17%, respectively) scales. Our analyses, which showed spatiotemporal patterns of maize and wheat yields and CWP across China, can be used to optimize agricultural production strategies in the context of maintaining food security.

## Abstract (summary)

**Table:** 

Measurement(s)	crop yield and crop water productivity
Technology Type(s)	remote sensing and machine learning

## Background & Summary

Crop water productivity (CWP), calculated as the ratio of crop yield to gross evapotranspiration (ET), is a quantitative indicator of agricultural performance^1^ that may be used to assess the impact of agri-environment and crop management strategies on crop growth^2,3^. Thus, accurate measurement of crop yield and ET as components of CWP is important in agricultural production decision-making and management of water resources^4^.

Methods that measure ET, such as lysimeter devices^5^ and the eddy covariance technique^6^, and approaches to its estimation, such as the energy balance Bowen ratio^7^ and the Penman-Monteith algorithm^8,9^, have tended to be used in point-scale and small area-scale studies^10^, while crop yield has generally been measured using quantitative field-based sampling, qualitative farmer or expert estimates, and micrometeorological measurements^1^. Policy-driven management of agricultural production often requires regional-scale, high spatial resolution monitoring of yield and ET; however, conventional methods and approaches to ET measurement and estimation are limited by low levels of efficiency and a lack of suitability for regional scale studies. Thus, remote-sensing technology has been adopted as an alternative data source for regional-scale, high spatial resolution estimates of ET, including in the Surface Energy Balance Algorithm for Land^11,12^, the Surface Energy Balance System^13^, the Two-source Energy Balance method^14^, and improved Penman-Monteith^15,16^ and Priestley-Taylor^17^ algorithms, where the widely used MOD16 ET product, generated using the improved Penman–Monteith method, has been shown to have good levels of accuracy^18,19^.

Estimates of remotely sensed (RS) crop yields derive from data assimilation (DA) in crop models^20–23^ or regression analysis of RS indicators (RSIs)^1,24^. In general, the DA approach has been applied over a wide range of crops and land surface and environment conditions^23^, for example, Jin, *et al*.^25^ assimilated RS data from RADARSAT-2 and HJ-1A/B into an AquaCrop model to estimate wheat yields (*R*^2^ = 0.42). However, performance of crop models is limited by complexity and uncertainty of input parameters, such as soil properties, meteorological data, crop cultivars, and management practices, that negatively affect simulation processes and cause larger errors in crop yield estimates^26^. In contrast, approaches that use RSI are based on fitted relationships, which tend to be nonlinear^24,27^, between *in-situ* measurements of yield and indicators, such as vegetation indices (VIs), ET, and gross primary productivity (GPP)^28–30^. These approaches have been widely used, due to their simplicity and efficiency; for example, Noland, *et al*.^31^ found 81−90% of the variation in alfalfa yields was explained by VIs calculated from multispectral data and Cao, *et al*.^32^ found the combination of the enhanced vegetation index (EVI) with deep-learning algorithms accounted for 71% of the variation in winter wheat yields. Machine-learning algorithms are well suited for dealing with nonlinear heteroscedastic problems and are used for efficient data processing and data mining^33,34^, and algorithms, such as support vector regression^35^, random forest (RF) regression^36^, and artificial neural networks^35^, have been used successfully to analyze agricultural RS data. For example, Maimaitijiang, *et al*.^35^ analyzed multimodal data (canopy texture and structure, spectra and temperature) collected by unmanned aerial vehicles (UAV) using machine-learning algorithms to estimate field-scale soybean yields, while Johansen, *et al*.^37^ leveraged multi-spectral UAV data and a RF model to prediction tomato phenotype yield and biomass.

The distribution of water resources across China is heterogenous, with particular areas of scarcity in the northwest^38^, and nationally, agricultural production accounts for 60−65% of water consumption^39^. Maize and wheat are staple dryland crops in China, with areas of cultivation of 41.3 × 10^6^ and 23.7 × 10^6^ ha, respectively, in 2019, so the accurate estimation of CWP at high spatial resolution is essential for ensuring sustainable agricultural production and water resource management in the context of maintaining food security. Currently, understanding of CWP of key food security crops in China is lacking, therefore, the aim of this study was to estimate CWP of maize and wheat across China at a high level of spatiotemporal resolution, based on multiple remote sensing indicators and combined ensemble machine learning and RF algorithms. Specifically, our objectives were to: (1) evaluate the accuracy of the MOD16 ET product in the estimation of crop water consumption; (2) test the accuracy of estimates of CWP based on RS-EVI and combined machine learning and RF algorithms; and, (3) quantify spatiotemporal patterns of crop yield and CWP across China.

## Methods

### Study area

**Fig. 1:**
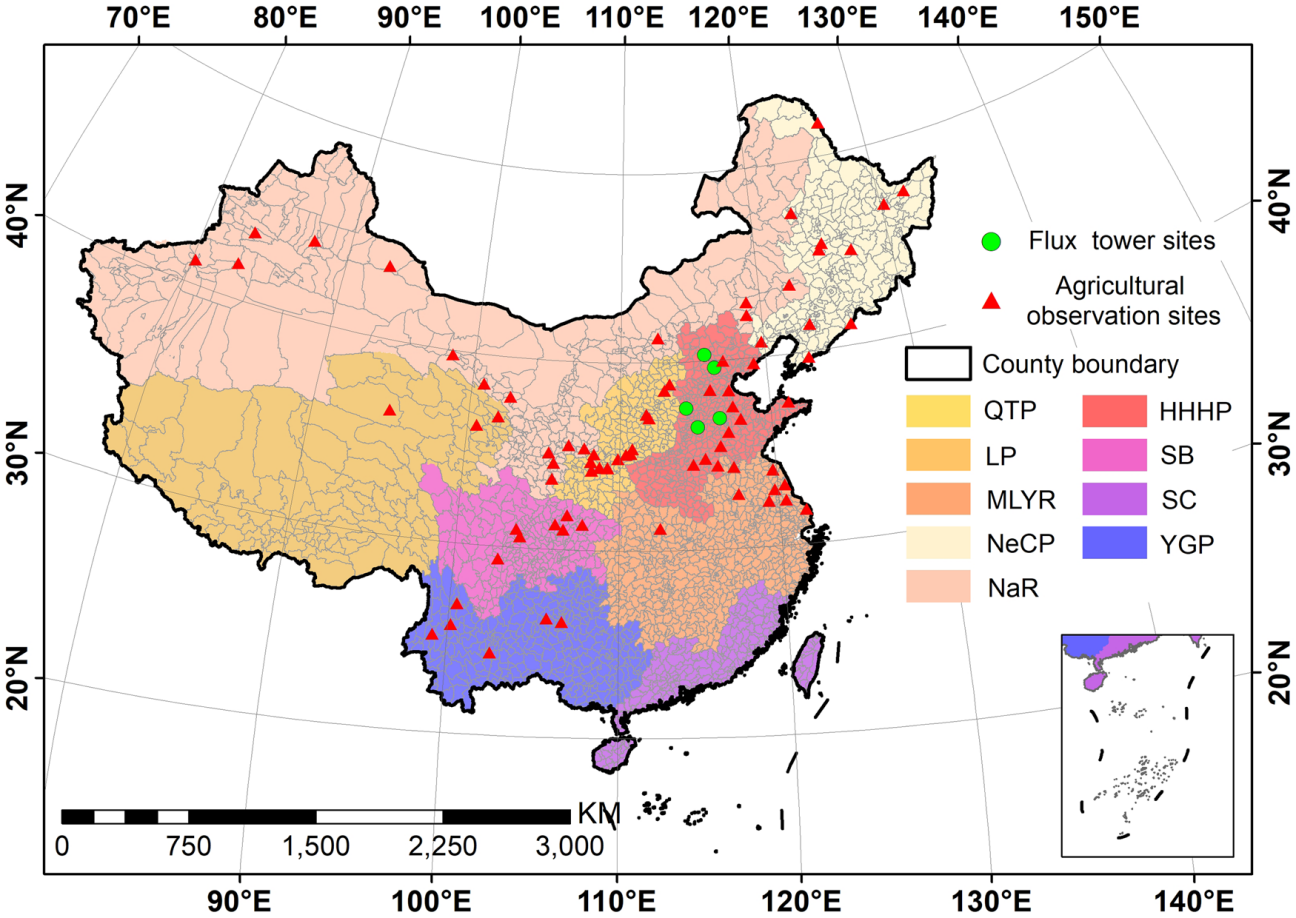
Study area and study sites by agricultural production region. QTP: Qinghai Tibet Plateau; HHHP: Huang-Huai-Hai Plain; LP: Loess Plateau; SB: Sichuan Basin; MLYR: Middle-lower Yangtze Plain; SC: Southern China; NeCP: Northeast China Plain; YGP: Yunnan-Guizhou Plateau; and, NaR: Northern arid and semiarid region.

China (3°31′00″–53°33′47″N, 73°29′59.79″–135°2′30″E) covers a land area of approximately 9.6 × 10^6^ km^2^ that is largely dominated by temperate climate conditions, with tropical climate conditions prevailing over a smaller relative area. The study area comprised the Qinghai Tibet Plateau (QTP), Huang-Huai-Hai Plain (HHHP), Loess Plateau (LP), Sichuan Basin (SB), Middle-lower Yangtze River Plain (MLYR), Northeast China Plain (NeCP), Yunnan-Guizhou Plateau (YGP), and the Northern arid and semiarid region (NaR) regions of agricultural production, but excluded Southern China (SC) due to the small areas of cultivation of maize and wheat^40^ (Fig. 1).

### Study parameters and data sources

#### Cropland map

**Fig. 2:**
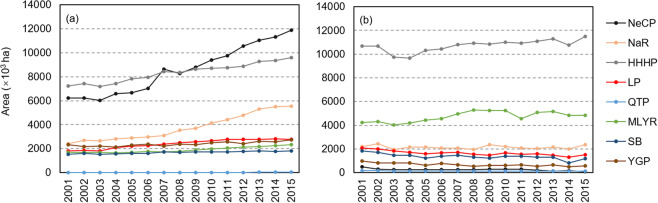
Cultivation areas of maize (**a**) and wheat (**b**) in China over the period 2001−15. QTP: Qinghai Tibet Plateau; HHHP: Huang-Huai-Hai Plain; LP: Loess Plateau; SB: Sichuan Basin; MLYR: Middle-lower Yangtze Plain; SC: Southern China; NeCP: Northeast China Plain; YGP: Yunnan-Guizhou Plateau; and, NaR: Northern arid and semiarid region.

We used cultivation area, yield, and CWP data for maize and wheat from 2001 to 2015. Data for cultivation area of maize and wheat were obtained from the 1-km National Land Cover Dataset (NLCD) (http://www.resdc.cn; Fig. 2) and generally showed an increase over the study period in most regions, where area of maize cultivation was greatest in NeCP and HHHP and area of wheat cultivation was greatest in HHHP.

#### Input variables

We selected seven indicators of crop yield (GPP; ET; land surface temperature, Ts; leaf area index, LAI; and, soil content of clay, sand, silt) as model inputs to estimate maize and wheat yield. Crop phenology data (annual at 1-km) were obtained from the ChinaCropPhen1km dataset^41,42^ that comprises Julian day (day of the year, DOY) of the main crop growth stages: from V3 in maize (the third leaf is fully expanded) to maturity, and from emergency (spring wheat) or green up (winter wheat) to maturity in wheat.

Data for Ts and **c**rop ET, GPP, and LAI were obtained from MOD11A2 Ts products, MOD16A2 ET products, MOD17A2 GPP products, and MOD15A2 LAI products, respectively, for regular 500-m grid cells aggregated to 1 km, to harmonize with the 1-km resolutions of the NLCD and ChinaCropPheno datasets, for the global vegetated land surface at an 8-d composite. Soil clay, silt, and sand content data were obtained from the 1:1 million soil type map and soil profile data were obtained from the Second China Soil Survey^43^; all soil data were at a spatial resolution of 1 km.

#### *In situ* crop yield

Crop yield data across the study period at the administrative county level were obtained from the China Rural Statistical Yearbook in the National Bureau of Statistics of China (NBSC, http://www.stats.gov.cn/), with gaps of several years in parts of some counties, and outliers were identified and excluded if they were outside the range of biophysical attainable yields (maize: <500 kg/ha or >15,000 kg/ha; wheat: <500 kg/ha or >13,000 kg/ha), or they were greater or less than 3 SD from the study period average, or derived from counties with >10,000 ha of planting area^32,44,45^. As a result of this filtering process, our dataset comprised 1981 and 2487 records of maize and wheat yields, respectively. Pixel-level crop yield data, derived from the National Meteorological Data Center of China^41^, were measured at 12 (in which, a total of 9 sites recorded two year’s samples and others only recorded one year’s sample) and 23 (in which, a total of 11 sites recorded three year’s samples, 6 sites recorded two year’s samples and others only recorded one year’s sample) study sites for maize and wheat, respectively, and at 42 study sites (only recorded one year’s sample) for both crops in a rotation. In summary, a total of 63 maize yield samples and 103 wheat yield samples were available for validation. It should be noted that the crop yield at county level and pixel level were recorded based on the harvested and measured grain yield, in which the maize yield was converted at the moisture of 14% and wheat yield was at 12.5%.

#### Flux tower observations

We derived EC data from ChinaFLUX recording stations located in maize and wheat crops in Daxing, Guantao, Huailai, Luancheng, and Yucheng for MOD16 ET assessment (Fig. 1), where ET was cumulated over 8-d periods, to harmonize with the MOD16 ET product temporal resolution (8-day composite). Table 1 shows the main information and sources of all data used in this study.

**Table 1:** Data types, spatiotemporal resolution, and sources.

Data type	Temporal resolution	Spatial resolution	Source
Evapotranspiration (ET)	8-day composite	500 m (Aggregated to 1 km)	NASA, MOD16A2 ET product (http://ladsweb.modaps.eosdis.nasa.gov)
Gross primary productivity (GPP)	8-day composite	500 m (Aggregated to 1 km)	NASA, MOD17A2 GPP product (http://ladsweb.modaps.eosdis.nasa.gov)
Surface temperature (Ts)	8-day composite	500 m (Aggregated to 1 km)	NASA, MOD11A2 Ts product (http://ladsweb.modaps.eosdis.nasa.gov)
Leaf area index (LAI)	8-day composite	500 m (Aggregated to 1 km)	NASA, MOD15A2 LAI product (http://ladsweb.modaps.eosdis.nasa.gov)
Soil properties	n/a	1 km	Resource and Environment Science and Data Center, Chinese Academy of Science (http://www.resdc.cn)
Phenology information	Yearly	1 km	ChinaCropPhen1km^41^ (10.6084/m9.figshare.8313530)
Cultivated-land layer	Yearly	1 km	Resource and Environment Science and Data Center, Chinese Academy of Science (http://www.resdc.cn)
Recorded yield (regional-scale)	Yearly	County-level	China Rural Statistical Yearbook, National Bureau of Statistics of China (http://www.stats.gov.cn)
Measured yield	Yearly	Point-scale	National Meteorological Data Center of China (http://data.cma.cn)
Flux tower observed data	Daily (Cumulated to eight days)	Point-scale	ChinaFLUX (http://www.chinaflux.org)

### Estimation of crop water productivity

#### Model process of evapotranspiration and yield

**Fig. 3:**
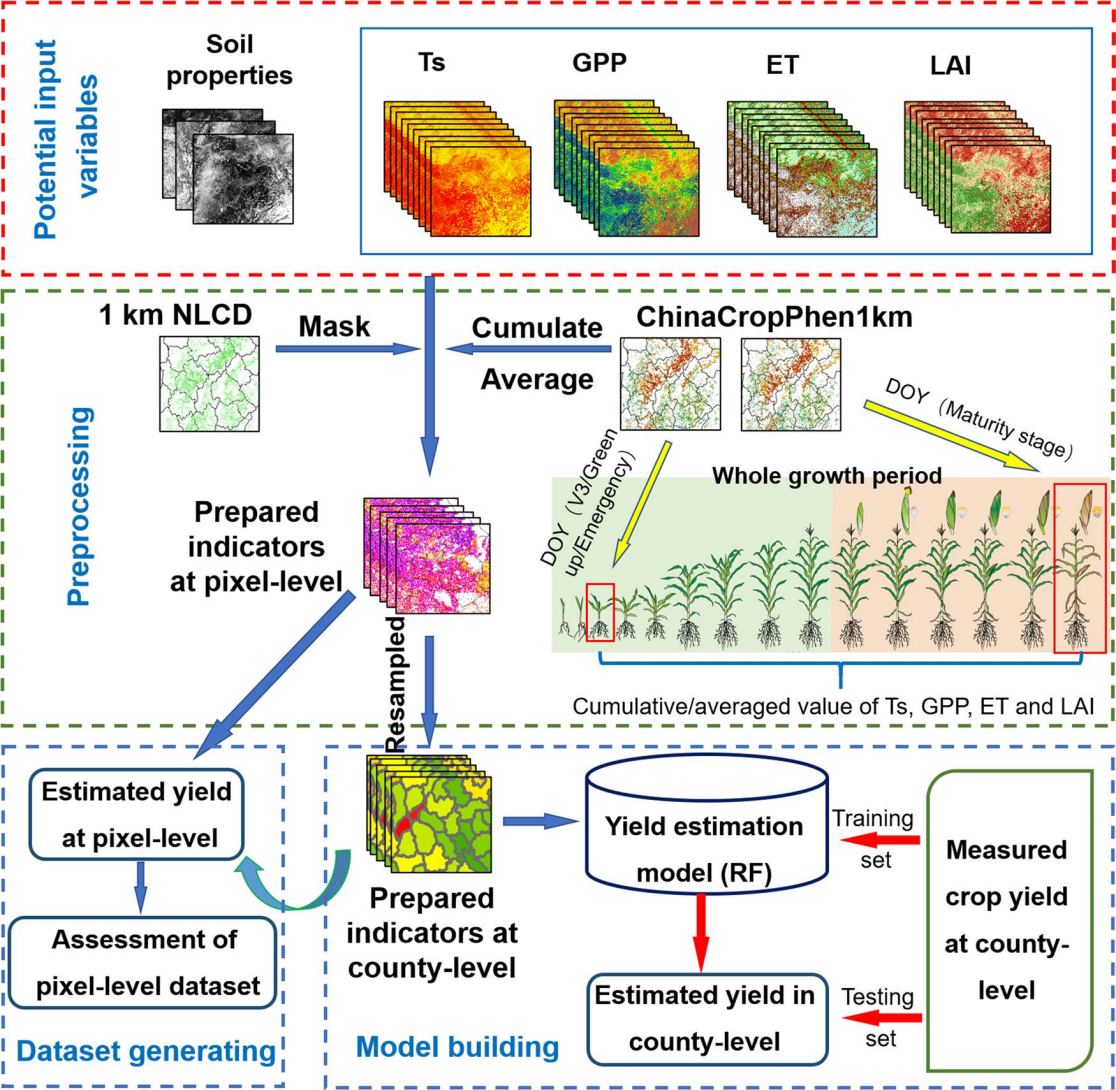
Schematic of data preprocessing, model construction, and generation of datasets for estimation of maize and wheat yields using RF and yield indicators.

Crop ET was derived from the MOD16 ET product, using an improved Penman-Monteith algorithm^15,16^ and crop yields were estimated using the Random Forest (RF) regression algorithm. The steps for generating the crop yield dataset are as follows:

1. Collecting the input variables: ET, GPP, LAI, Ts and three soil properties datasets. All the variables were resampled to 1 km spatial resolution by using Nearest algorithm^46^.
2. Using the 1 km National Land Cover Dataset (NLCD) to mask the seven input variables.
3. Using the 1 km ChinaCropPheno dataset to calculate the cumulative value of ET, GPP and Ts and the averaged value of LAI from the V3 stage of maize (emergency or green up stage of wheat) to maturity stage.
4. Statistic the seven indicators processed in (2) and (3) to county-level to match the annual crop yield from National Bureau of Statistics of China (NBSC).
5. Using RF to fit the seven indicators in county-level with the crop yield. In which, the 80% of the county-level maize yield samples were randomly selected for training the model estimates of yield, to ensure reliability, and the remaining 20% of samples were used to validate accuracy of the estimates. Model training data should contain maximum and minimum yield values. Given temperature^47^, GPP^48^, LAI^49^, and ET^50^ affect crop yield, they were input to the model individually and in combination, with effects of soil clay, sand, and silt content held as constant, to compare levels of accuracy of yield estimates and build the optimal model^46^.
6. After optimal model training for yield estimation had been completed, the input indicators at pixel-level resolution (processed in (2) and (3)) were directly input to generate pixel-level annual crop yield datasets, at a spatial resolution of 1 km. Using the point-scale crop yield data derived from the National Meteorological Data Center of China to assess the generated dataset. See Fig. 3 for workflow of data preprocessing, model construction, and generation of datasets.

#### Crop water productivity definition

We defined CWP (kg/m^3^) of maize and wheat as the ratio of yield to cumulative ET (Eq. 1):

1 $$CWP=\frac{Yield}{\sum ET}$$

where crop yield (kg/ha) was estimated by the proposed model; cumulative ET (mm) is across the main crop growth stage. In terms of the spatial difference of crop phenology, the cumulative ET was calculated using the ET from V3 stage of maize (emergency or green up stage of wheat) to maturity, which is the main period of crop growth stage. Therefore, it should be the cumulative ET in this study will less than other studies which were calculated in the whole crop growth period^4^.

#### Random forest algorithm

Random Forest (RF) regression algorithm is widely used ensemble learning method by combining multiple decision trees, where each regression tree represents a set of restrictions or conditions on indicators of the target variable; in this study, the variable is county-level crop yield. The RF algorithm begins with subsamples randomly selected from the training set, and then the regression tree is fitted to the subsamples; the final modeled value is the average across all trees. The details of RF can be referred to the study of Breiman^51^. In this study, the two important parameters: tree numbers and the randomly sampled potential variables in each split, were set as 100 and 4 by debugging and referring other studies^52^.

The RF algorithm has been shown to be effective in coping with over-fitting^53^, performs well in multiple regressions, and has been widely used in the analysis of RS data^32,35,52,54,55^.

### Assessment of model input and output accuracy

#### Evapotranspiration dataset

The EC method of estimating ET measures λET (latent heat flux) from covariance in heat and moisture fluxes, with vertical velocity using rapid response sensors at frequencies typically equal to or greater than 10 Hz, and is regarded as the most effective method for the estimation of ET^10^. The energy balance closure issue, which indicates the sum of sensible heat (H), λET and soil heat flux (G), is not equal to net radiation (Rn), is frequently found in the EC method, so values measured using this system value should be filtered and corrected. Here, data with energy balance closure ratios (ECR, Eq. 2) <80% were not selected for validation^56^ and the remaining data with ECR >80% were corrected using the Bowen ratio energy balance correction (Eq. 3)^57^.

2 $$ECR=\frac{H+\lambda ET}{Rn-G}$$

3 $$\lambda E{T}_{cor}=\frac{Rn-G}{H+\lambda ET}\times \lambda ET$$

where *Rn*, *G*, *H* and *λET* are values measured using the EC system, and *λET*_*cor*_ is the corrected value. To ensure reliable evaluation, the pixel value at the flux tower location (area: 1 × 1 km) was extracted for comparison with the measured value^19^.

#### Estimated yield

We used county-level empirical yield data in the model for yield estimation, where 20% of the samples (maize N = 396; wheat N = 497) were used for regional-scale validation of crop yields and empirical pixel-level yield data, obtained from the 12 maize, 23 wheat, and 42 mixed sites, were used to validate estimated yields at the point-scale. Each yield measurement site comprised data recorded over one or multiple years, and overall, our dataset comprised 63 maize and 103 wheat yield samples at the point-scale; pixel values (1 km) of estimated crop yields at these measurement sites were directly compared with their corresponding measured values.

#### Model performance

We calculated the adjusted coefficient of determination (*R*^2^), root-mean-square error (RMSE), relative root-mean-square error (rRMSE), and mean bias error (MBE), following Jin *et al*. (2020), to quantify model performance:

4 $${R}^{2}=1-\left(1-\frac{\mathop{\sum }\limits_{i=1}^{n}{\left({M}_{i}-\overline{M}\right)}^{2}}{\mathop{\sum }\limits_{i=1}^{n}{\left({O}_{i}-\overline{O}\right)}^{2}}\right)\frac{n-1}{n-m-1}$$

5 $$RMSE=\sqrt{\frac{1}{n}\mathop{\sum }\limits_{i=1}^{n}{\left({M}_{i}-{O}_{i}\right)}^{2}}$$

6 $$rRMSE=\frac{RMSE}{\overline{O}}\times 100 \% $$

7 $$MBE=\frac{1}{n}\mathop{\sum }\limits_{i=1}^{n}\left({M}_{i}-{O}_{i}\right)$$

where *M* and *O* are the estimated and recorded/measured value (ET or yield), respectively, *n* is the number of samples, and *m* is the number of variables.

#### Spatial autocorrelation analysis

Spatial patterns of crop yield are affected by spatiotemporal variations in soil properties, climate, land-use change, diseases, and management practices^58^, so heterogeneity and dependency of crop yield may similarly vary spatially, particularly over large areas^35^. While assumptions of location invariance and spatial independence have been applied to yield estimates^59,60^, they may lead to inaccurate model estimates without spatial variation and autocorrelation analysis^58^. To cope with this issue, we used Global Moran’s *I* (Moran^61^, which ranges from −1 to 1, to examine spatial autocorrelations between model yield estimate errors^35,62^ that were calculated as the difference between estimated and measured yields at the county level. Global Moran’s *I* represents the spatial autocorrelation of errors in estimates of yield or the degree of clustering^63^ and it has been used widely in the evaluation of model spatial performance^64,65^. In this study, a Global Moran’s *I* of zero indicates a random spatial distribution, while a near zero value indicates that errors in the estimates of yield were randomly distributed, where higher randomness tends to indicate better model performance over space. Global Moran’s *I* was calculated as follows:

8 $$I=\frac{n\times {\sum }_{i=1}^{n}{\sum }_{j=1}^{n}{\omega }_{ij}\left({x}_{i}-\overline{x}\right)\left({x}_{j}-\overline{x}\right)}{S\times {\sum }_{i=1}^{n}\left({x}_{i}-\overline{x}\right)}$$

where *n* is number of counties; *ω*_*ij*_ is the weight matrix between counties *i* and *j*, with a value of 1 or 0 when the two counties are adjacent or nonadjacent, respectively; *x*_*i*_ and *x*_*j*_ are the difference between estimated yield and recorded yield of counties *i* and *j*, respectively; and, *S* is the sum of *ω*_*ij*_.

Model performance, based on R^2^, rRMSE and Moran’s *I* across input single and combined indicators, was tested using one-way analysis of variance (ANOVA) at *P* < 0.01 in SPSS (Version 21, IBM Corp., Armonk, US). Similarly, differences in crop yield and CWP among the eight agricultural production regions were tested using ANOVA.

## Data Records

The dataset that was generated using random forest regression and multiple remotely sensing indicators, at a spatial resolution of 1 km and a yearly temporal resolution, which can be used for optimizing agricultural production strategies and water resources management, etc. The crop yield and water productivity dataset for China is distributed under a Creative Commons Attribution 4.0 International license. The dataset is named ChinaCYWP and consists of 15 years of data, with the format of TIF. More information and data are freely available from the Zenodo repository at 10.5281/zenodo.5121842^66^.

## Technical Validation

### Validation of evapotranspiration dataset

**Fig. 4:**
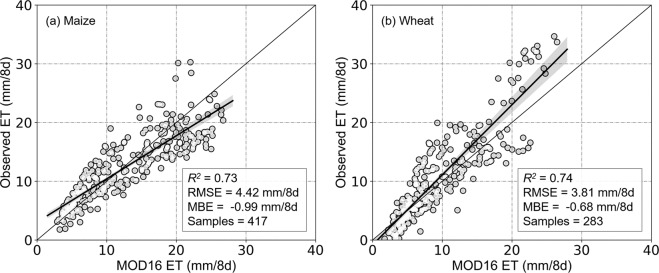
Validation of MOD16 ET products for (**a**) maize and (**b**) wheat. Note: *R*^*2*^ indicates coefficient of determination, RMSE indicates root-mean-square error and MBE indicates mean bias error.

Crop rotations at the five EC flux measurement stations comprised maize-wheat rotations, and we used the EC estimates of ET to validate MOD16 estimates of ET (Fig. 4). For maize, MOD16 estimates of ET varied from 4.18 to 27.51 mm/8d (*R*^2^ = 0.73; RMSE = 4.42 mm/8d), while for wheat, ET estimates varied from 1.39 to 26.32 mm/8d (*R*^2^ = 0.74; RMSE = 3.81 mm/8d). In general, MOD16 estimates of crop ET were lower than observed EC estimates of ET (maize MBE = −0.99 mm/8d; wheat MBE = −0.68 mm/8d).

In addition to the MOD16 ET product, several other ET products, such as Global Land Evaporation Amsterdam Model, GLEAM^67^, Global Land Data Assimilation System, GLDAS^68^, and Evapotranspiration-Energy Balance, ET-EB^69^ products, generated by different algorithms have been evaluated in previous studies^19,70,71^. Algorithms for the estimation of RS ET tend to be complementary, with contrasting strengths and weaknesses^72^; for example, the spatiotemporal resolution (500 m and 8-d composite) of MOD16 is finer than other ET products, including GLEAM (0.25° and daily), GLDAS (0.25° and monthly), and ET-EB (0.1° and daily), and is more appropriate for the generation of crop yield and CWP data at 1-km spatial resolution. As a result, we found that MOD16 yielded an acceptable level of accuracy for describing the ET of maize and wheat. Previous research has also demonstrated the greater estimate accuracy of MOD16 products, including Velpuri, *et al*.^19^, who concluded that accuracy of MOD16 for estimates of cropland flux tower data was greater than that of SSEBop, while Khan, *et al*.^73^ similarly found that accuracy of MOD16 in cropland was greater (bias: 0.22 mm/8 d) than that of GLDAS and GLEAM (4.32 and 5.35 mm/8d, respectively). Although validation of flux tower data represent a useful method for ET measurement^10^, uncertainties remain, including large error size (10–30%) in eddy covariance flux tower data^70,74^ and mismatches between flux tower footprint and RS information caused by effects of wind direction, atmospheric stability, and surface type^75^.

### Validation of model yield estimates

#### Regional-scale

**Fig. 5:**
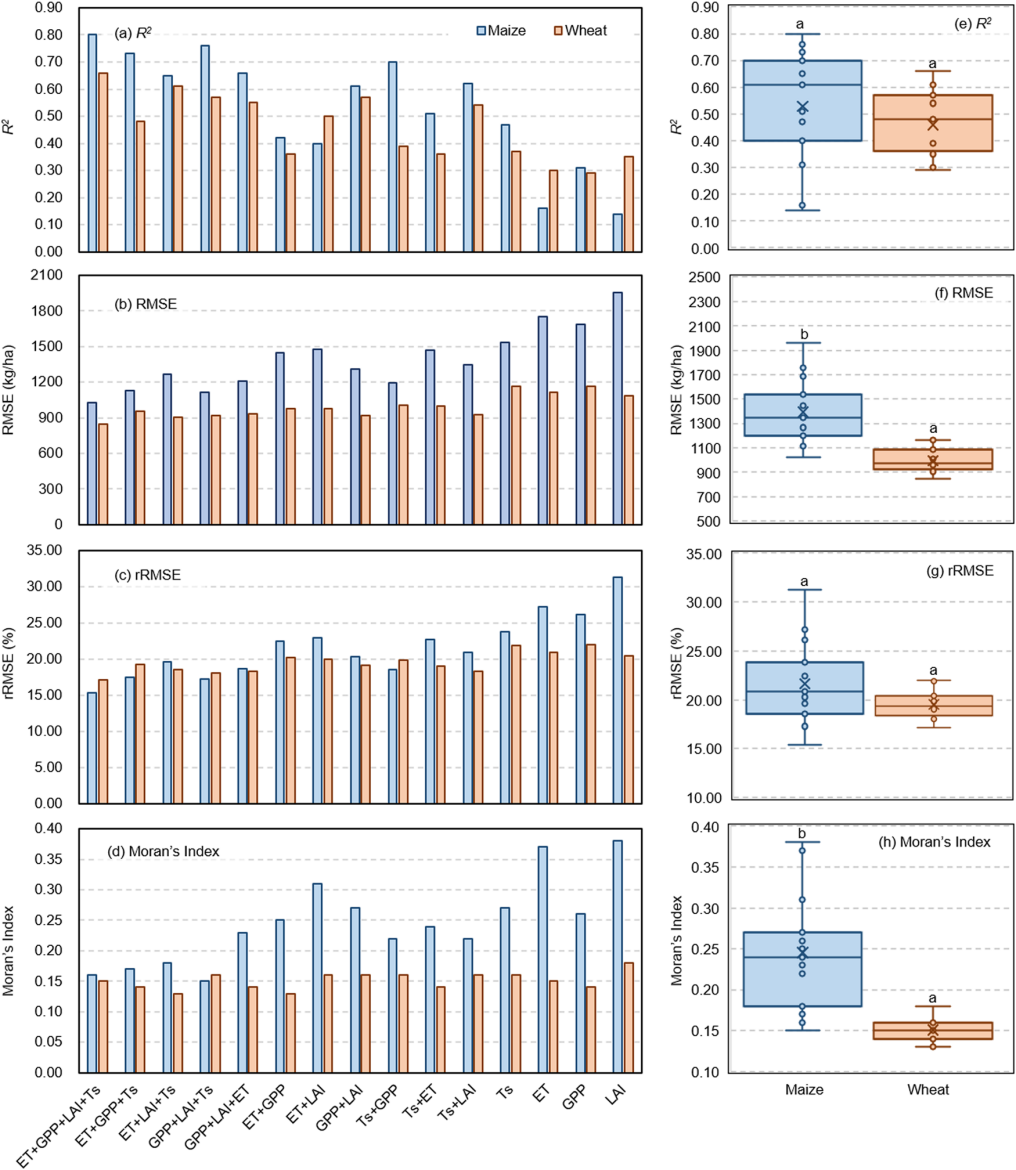
Model estimates of crop yield based on inclusion of single and combined indicators: histogram of (**a**) *R*^*2*^, (**b**) RMSE, (**c**) rRMSE, and (**d**) Moran’s *I*; distribution of median and range (±95% CI) of (**e**) *R*^*2*^, (**f**) RMSE, (**g**) rRMSE, and (**h**) Moran’s *I*. Different letters indicate differences in accuracy of crop model yield estimates at *P* < 0.01.

In general, the accuracy of maize and wheat yield estimates improved with increasing number of input indicators, with four indicators accounting for the greatest amount of variation in yield estimates (maize *R*^2^ = 0.80, rRMSE = 15.36%; wheat *R*^2^ = 0.66, rRMSE = 17.17%), and while there were no differences in *R*^2^ and rRMSE indicators of model estimates between the two crops (*P* < 0.01), RMSE for maize (1025−1958 kg/ha) was larger than for wheat (845−1166 kg/ha) (*P* < 0.01) (Fig. 5). In general, Moran’s *I* decreased with increasing number of indicators included in the model (i.e., better spatial applicability), where it was lowest for maize with the inclusion of four indicators (*I* = 0.16) and lowest in wheat when ET, LAI, and Ts were included (*I* = 0.13) (Fig. 5).

**Fig. 6:**
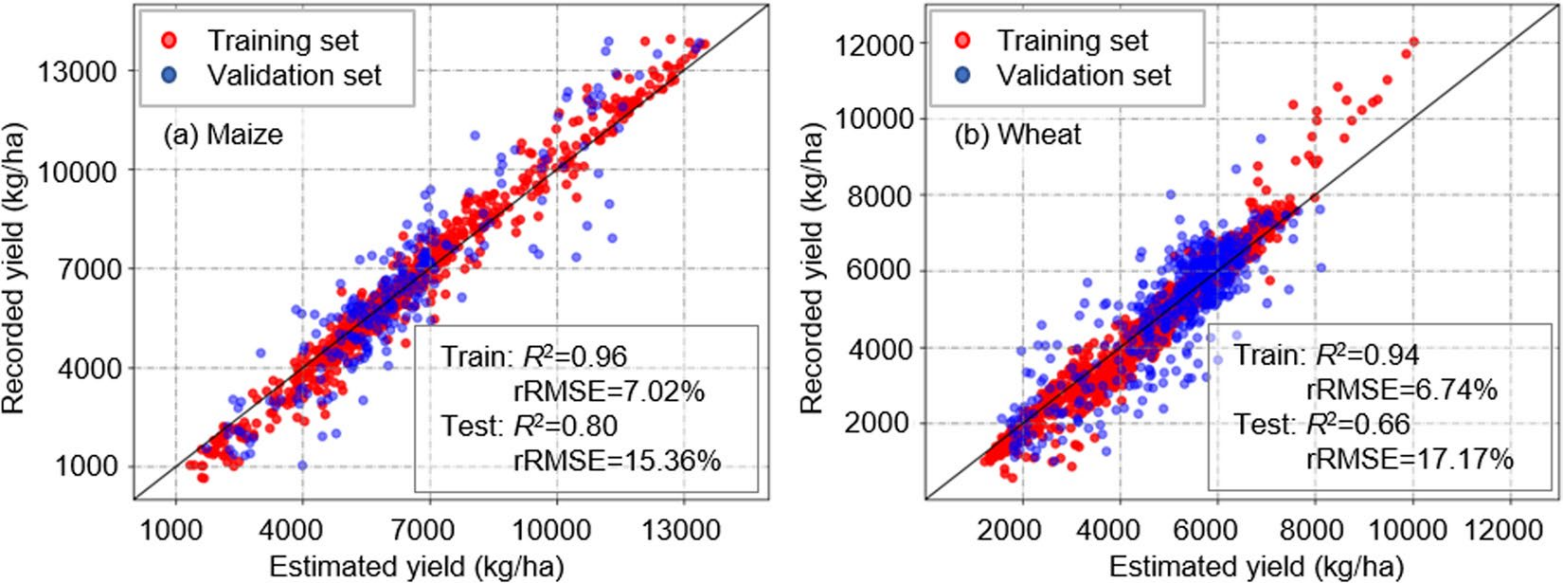
Regional-scale validation of estimated maize (**a**) and wheat (**b**) yields based on model inclusion of GPP, ET, Ts, and LAI. Note: *R*^*2*^ indicates coefficient of determination and rRMSE indicates relative root-mean-square error.

Overall, inclusion of four indicators led to best estimates of maize (*R*^2^ = 0.80; rRMSE = 15.36%) and wheat (*R*^2^ = 0.66; rRMSE = 17.17%) yields (Fig. 6). Thus, the pixel-level crop yield dataset was generated using the four indicators.

#### Point-scale

**Fig. 7:**
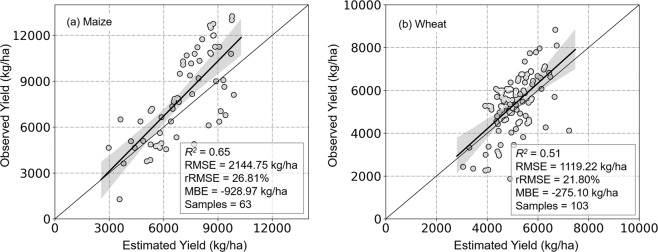
Point-scale validation of estimated maize (**a**) and wheat (**b**) yields based on model inclusion of GPP, ET, Ts, and LAI.

We found pixel-scale estimates of maize and wheat yields, based on point-scale yield data, were similar (maize: *R*^2^ = 0.65, RMSE = 2144.75 kg/ha, rRMSE = 26.81%; wheat: *R*^*2*^ = 0.51, RMSE = 1119.22 kg/ha, rRMSE = 21.80%), while model performance was less accurate than for regional-scale estimates, with underestimates (MBE) of maize and wheat crop yield, compared with empirical data, of −928.91 and −275.10 kg/ha, respectively (Fig. 7).

#### Summary

Approaches for crop yield estimation based on RS data^29,32,35,37,76,77^ tend to use single or multi-phase RS images to describe crop growth status and estimate yield; for example, Maimaitijiang, *et al*.^35^ used single-phase UAV images (multi-sensors) at the start of the pod stage of soybean to estimate yield. However, given the status of each stage of the entire growth period may contribute to crop final yield, phenological information, such as that provided by crop growth stage indicators, is likely to be essential for accurate crop yield estimation. Indeed, Guo, *et al*.^78^ found the inclusion of phenology and climate data led to more accurate model estimates of rice yield in China (*R*^*2*^ = 0.33 and RMSE = 737 kg/ha). Remotely sensed data for yield estimation tends to be based on VIs, such as in the studies by Cao, *et al*.^32^ and Chen, *et al*.^77^, who used RS normalized difference vegetation index (NDVI) and a combination of NDVI, enhanced vegetation index (EVI), and soil adjusted vegetation index (SAVI), respectively, to estimate maize yield in China. Although physiological indicators of crop growth, such as GPP and ET, correlate with crop yield^48,50,79^, characterization of crop growth status by VIs may be limited, whereas relative indicators of temperature, such as growing degree days and effective accumulated temperature (EAT), have been shown to be associated with crop growth status and yield^80–82^. Of the single indicators used in this study, we found that cumulative Ts, which may be regarded as EAT without threshold filtering, explained most of the variation in maize yield (Fig. 5); in contrast, Maimaitijiang, *et al*.^35^ found that Ts were poor predictors of soybean yield, possibly due to the use of single-phase images.

**Fig. 8:**
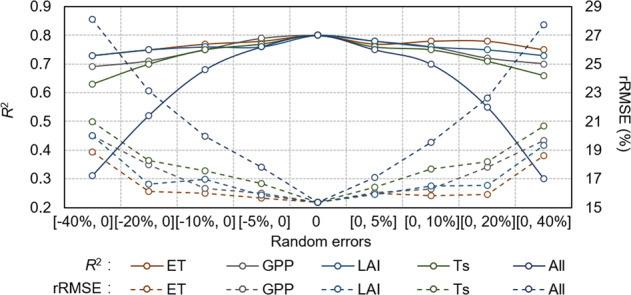
Sensitive analysis of the effect of input variables to yield prediction.

In order to further explore the influence by the accuracy of the input indicators to model performance, a sensitive analysis was conducted by taking the maize yield estimation as an example, i.e., a random error was artificially set in each indicator or multi-indicators, and the changes in performance were analyzed. The sensitive analysis method was referred to Cheng, *et al*.^39^ and Long, *et al*.^83^. The results were showed in Fig. 8. In general, the model still performed good (*R*^2^ > 0.62 and rRMSE < 20%) when only one indicator had errors, even if a random error between 0 to 40% (−40% to 0) was set. The model results changed the most when the errors were existed in Ts. But these differences among the four indicators was small. However, when the four indicators all had errors, the model performance changed a lot. The *R*^2^ was decreased to 0.30 when random errors of 0 to 40% were existed in the four indicators and rRMSE was increased to 28.12% when random errors of −40 to 0 were existed, which were the worst situation. As reported in previous studies, MODIS products have errors to different extents. For example, MOD16 ET product showed approximately 15–30% errors in China^39^. MOD17 GPP product has been evaluated by Liu, *et al*.^84^ and showed *R*^2^ varied from 0.21 to 0.90 in China. Be that as it may, the proposed method still performed an acceptable robustness and tolerance when confronted to the uncertainties of indicators accuracy. Which was likely contributed by the correlations among indicators, i.e., when the information of a specific indicator was loss caused by the accuracy errors, the other indicators which have strong correlation, may fill this information gap.

Overall, our proposed model for estimation of maize and wheat yields performed with good accuracy at county-level (rRMSE: 15.36 and 17.17%, respectively) and pixel-level validation (rRMSE: 26.81 and 21.80%, respectively). These levels of accuracy are comparable to, or greater than previous studies^29,32,77^ and, although the accuracy of the yield estimates improved with increasing number of input indicators, we found the accuracy of wheat yield estimates was lower than that for maize, possibly as a result of duplicated information among some indicators. We note a lower performance of model estimates of maize and wheat yield performance at the pixel-level than county-level, possibly due to model training by county-level yield data and potential differences in data measurement protocols.

Many scholars have made efforts to estimate CWP. Bastiaanssen and Steduto^4^ estimated the average value of global maize CWP by using WATPRO model as 2.25 ± 0.94 kg/m^3^; Edreira, *et al*.^85^ estimated that the CWP of maize in Africa was 1.8 kg/m^3^ and that in Europe was 2.9 kg/m^3^ by using meteorological data and crop models. Li, *et al*.^86^ estimated the CWP of maize in Hetao irrigated area as 2.59–3.34 kg/m^3^ by using the AquaCrop model. In comparing, the CWP estimated in this study presented relative higher than others (4.14 ± 1.62 and 4.78 ± 2.43 kg/m^3^ for maize and wheat, respectively), three causes were discussed as follows: (1) as proved in Section 4.1, MOD16 presented a certain underestimation of crop ET, in which, MBE was −0.99 mm/8d for maize and −0.68 mm/8d for wheat; (2) the cumulative ET of the crop growth period in this study was calculated using the ET from V3 stage of maize (emergency or green up stage of wheat) to maturity stage, which was shorter than the whole crop growth period The short time period also caused the lower accumulated ET; (3) this study was conducted covering whole China planting area of maize and wheat, including rainfed and spring maize planting area, which lead the lower ET than irrigated area and summer maize planting area^85^. In general, lower ET estimation caused the higher CWP. Despite all this, the CWP dataset generated in this study presented a certain accuracy and comparability of spatial and temporal.

Although we found that maize and wheat ET and yield were good predictors of observed CWP, direct verification of RS CWP is difficult^1^, because *in situ* benchmark values for CWP tend not to be available^4^; however, given some calculations of CWP have been based on GPP, rather than crop yield, it is possible to directly evaluate estimates using EC flux tower observations^56,87^. Even though we found separate validation of the two CWP components to be acceptable, the uncertainties from error propagation should not be ignored and we recommend further studies to identify improved methods for the validation of gridded CWP datasets.

## Data Availability

The codes we developed for crop yield computation and crop yield dataset generation are available at 10.5281/zenodo.6444614^88^. The code was programmed using Python 3.9. In this code, we used the sklearn library for calling machine learning algorithm and GDAL library for raster data reading and processing. Moreover, the band calculation tool of ArcGIS 10.4 was used for crop water productivity dataset generation.

## References

[1] Blatchford ML, Mannaerts CM, Zeng Y, Nouri H, Karimi P (2019). Status of accuracy in remotely sensed and *in-situ* agricultural water productivity estimates: A review. Remote Sensing of Environment.

[2] Geerts S, Raes D (2009). Deficit irrigation as an on-farm strategy to maximize crop water productivity in dry areas. Agricultural Water Management.

[3] Hellegers P, Soppe R, Perry C, Bastiaanssen W (2009). Combining remote sensing and economic analysis to support decisions that affect water productivity. Irrigation Science.

[4] Bastiaanssen, W. G. M. & Steduto, P. The water productivity score (WPS) at global and regional level: Methodology and first results from remote sensing measurements of wheat, rice and maize. *The Science of the total environment* **575**, 10.1016/j.scitotenv.2016.09.032 (2017). 10.1016/j.scitotenv.2016.09.032 27712867

[5] Seneviratne, S. I. *et al*. Investigating soil moisture–climate interactions in a changing climate: A review. *Earth Science Reviews* **99**, 10.1016/j.earscirev.2010.02.004 (2010).

[6] Hu, X., Shi, L., Lin, L. & Zha, Y. Nonlinear boundaries of land surface temperature–vegetation index space to estimate water deficit index and evaporation fraction. *Agricultural and Forest Meteorology* **279**, 10.1016/j.agrformet.2019.107736 (2019).

[7] Bowen IS (1926). The Ratio of Heat Losses by Conduction and by Evaporation from any Water Surface. Physical Review.

[8] Penman, H. L. Natural evaporation from open water, hare soil and grass. *Proceedings of the Royal Society of London. Series A, Mathematical and physical sciences* **193**, 10.1098/rspa.1948.0037 (1948). 10.1098/rspa.1948.0037 18865817

[9] Monteith, J. L. Evaporation and environment. The stage and movement of water in living organisms. *Symp.soc.exp.biol.the Company of Biologists* (1965).

[10] Wang, K. & Dickinson, R. E. A review of global terrestrial evapotranspiration: Observation, modeling, climatology, and climatic variability. *Reviews of Geophysics* **50**, 10.1029/2011RG000373 (2012).

[11] Bastiaanssen WG (1998). A remote sensing surface energy balance algorithm for land (SEBAL) Part 1: Fomulation. Journal of hydrology.

[12] Bastiaanssen, W. G. M. *et al*. A remote sensing surface energy balance algorithm for land (SEBAL) Part 2. Validation. *Journal of Hydrology* **212**, 10.1016/S0022-1694(98)00254-6 (1998).

[13] Su Z (2002). The Surface Energy Balance System (SEBS) for estimation of turbulent heat fluxes. Hydrology and Earth System Science.

[14] Norman, J. M., Kustas, W. P. & Humes, K. S. Source approach for estimating soil and vegetation energy fluxes in observations of directional radiometric surface temperature. *Agricultural and Forest Meteorology* **77**, 10.1016/0168-1923(95)02265-y (1995).

[15] Mu, Q., Heinsch, F. A., Zhao, M. & Running, S. W. Development of a global evapotranspiration algorithm based on MODIS and global meteorology data. *Remote Sensing of Environment* **111**, 10.1016/j.rse.2007.04.015 (2007).

[16] Mu Q, Zhao M, Running SW (2011). Improvements to a MODIS global terrestrial evapotranspiration algorithm. Remote Sensing of Environment.

[17] Fisher JB, Tu KP, Baldocchi DD (2008). Global estimates of the land–atmosphere water flux based on monthly AVHRR and ISLSCP-II data, validated at 16 FLUXNET sites. Remote Sensing of Environment.

[18] Kim, H. W., Hwang, K., Mu, Q., Lee, S. O. & Choi, M. Validation of MODIS 16 global terrestrial evapotranspiration products in various climates and land cover types in Asia. *KSCE Journal of Civil Engineering* **16**, 10.1007/s12205-012-0006-1 (2012).

[19] Velpuri, N. M., Senay, G. B., Singh, R. K., Bohms, S. & Verdin, J. P. A comprehensive evaluation of two MODIS evapotranspiration products over the conterminous United States: Using point and gridded FLUXNET and water balance ET. *Remote Sensing of Environment* **139**, 10.1016/j.rse.2013.07.013 (2013).

[20] Jin X (2016). Estimation of water productivity in winter wheat using the AquaCrop model with field hyperspectral data. Precision Agriculture.

[21] Felix R, Clement A, Igor S, Oscar R (2013). Using Low Resolution Satellite Imagery for Yield Prediction and Yield Anomaly Detection. Remote Sensing.

[22] Lu, Y. *et al*. Assimilation of soil moisture and canopy cover data improves maize simulation using an under-calibrated crop model. *Agricultural Water Management* **252**, 10.1016/j.agwat.2021.106884 (2021).

[23] Jin, X., Kumar, L., Li, Z., Feng, H. & Wang, J. A review of data assimilation of remote sensing and crop models. *European Journal of Agronomy* **92**, 10.1016/j.eja.2017.11.002 (2018).

[24] Weiss, M., Jacob, F. & Duveiller, G. Remote sensing for agricultural applications: A meta-review. *Remote Sensing of Environment* **236**, 10.1016/j.rse.2019.111402 (2019).

[25] Jin X (2017). Winter wheat yield estimation based on multi-source medium resolution optical and radar imaging data and the AquaCrop model using the particle swarm optimization algorithm. ISPRS Journal of Photogrammetry and Remote Sensing.

[26] Tao, F., Rötter, R. P., Palosuo, T., Díaz-Ambrona, C. G. H. & Schulman, A. H. Contribution of crop model structure, parameters and climate projections to uncertainty in climate change impact assessments. *Global Change Biology* **24**, 10.1111/gcb.14019 (2017). 10.1111/gcb.14019 29245185

[27] Jin X (2018). A review of data assimilation of remote sensing and crop models. European Journal of Agronomy.

[28] Anikó K (2018). Statistical modelling of crop yield in Central Europe using climate data and remote sensing vegetation indices. Agricultural and Forest Meteorology.

[29] Wang Y, Zhang Z, Feng L, Du Q, Runge T (2020). Combining Multi-Source Data and Machine Learning Approaches to Predict Winter Wheat Yield in the Conterminous United States. Remote Sensing.

[30] Franz, T. E. *et al*. The role of topography, soil, and remotely sensed vegetation condition towards predicting crop yield. *Field Crops Research* **252**, 10.1016/j.fcr.2020.107788 (2020).

[31] Noland RL (2018). Estimating alfalfa yield and nutritive value using remote sensing and air temperature. Field Crops Research.

[32] Cao, J., Zhang, Z., Luo, Y., Zhang, L. & Tao, F. Wheat yield predictions at a county and field scale with deep learning, machine learning, and google earth engine. *European Journal of Agronomy*, 126204, 10.1016/j.eja.2020.126204 (2021).

[33] Jacinta H, Kerrie M (2018). Statistical Machine Learning Methods and Remote Sensing for Sustainable Development Goals: A Review. Remote Sensing.

[34] Jin X, Liu S, Baret F, Hemerlé M, Comar A (2017). Estimates of plant density of wheat crops at emergence from very low altitude UAV imagery. Remote Sensing of Environment.

[35] Maimaitijiang M (2020). Soybean yield prediction from UAV using multimodal data fusion and deep learning. Remote Sensing of Environment.

[36] Hossein, A., Mohsen, A., Davoud, A., Salehi, S. H. & Soheil, R. Machine Learning Regression Techniques for the Silage Maize Yield Prediction Using Time-Series Images of Landsat 8 OLI. *IEEE Journal of Selected Topics in Applied Earth Observations Remote Sensing* **PP**, 1–15, 10.1109/JSTARS.2018.2823361 (2018).

[37] Johansen K (2020). Predicting Biomass and Yield in a Tomato Phenotyping Experiment Using UAV Imagery and Random Forest. Frontiers in Artificial Intelligence.

[38] Zhang, L., Ding, X., Shen, Y., Wang, Z. & Wang, X. Spatial Heterogeneity and Influencing Factors of Agricultural Water Use Efficiency in China. *Resources and Environment in the Yangtze Basin* **28**, 10.11870/cjlyzyyhj201904008 (2019).

[39] Cheng, M. *et al*. Satellite time series data reveal interannual and seasonal spatiotemporal evapotranspiration patterns in China in response to effect factors. *Agric. Water Manage*. **255**, 10.1016/j.agwat.2021.107046 (2021).

[40] Zhou, L. *Comprehensive agricultural regionalization in China*. (Agricultural Press of China, 1985).

[41] Luo Y, Zhang Z, Chen Y, Li Z, Tao F (2019). Figshare.

[42] Luo Y, Zhang Z, Chen Y, Li Z, Tao F (2020). ChinaCropPhen1km: a high-resolution crop phenological dataset for three staple crops in China during 2000–2015 based on leaf area index (LAI) products. Earth System Science Data.

[43] Song, D. *Second China Soil Survey*. (Chinese Science Press, 1979).

[44] Zhang T, Yang X, Wang H, Li Y, Ye Q (2014). Climatic and technological ceilings for Chinese rice stagnation based on yield gaps and yield trend pattern analysis. Global Change Biology.

[45] Chen Y, Zhang Z, Tao F (2018). Improving regional winter wheat yield estimation through assimilation of phenology and leaf area index from remote sensing data. European Journal of Agronomy.

[46] Cheng, M. *et al*. Combining multi-indicators with machine-learning algorithms for maize yield early prediction at the county-level in China. *Agricultural and Forest Meteorology* **323**, 10.1016/j.agrformet.2022.109057 (2022).

[47] Amir J, Sinclair T (1991). A model of the temperature and solar-radiation effects on spring wheat growth and yield. Field Crops Research.

[48] Prince SD, Haskett J, Steininger M, Wright SR (2001). Net Primary Production of U.S. Midwest Croplands from Agricultural Harvest Yield Data. Ecological Applications.

[49] Gilardelli C (2019). Downscaling rice yield simulation at sub-field scale using remotely sensed LAI data. European journal of agronomy.

[50] Shakoor R, Hassan MY, Raheem A, Wu Y-K (2016). Wake effect modeling: A review of wind farm layout optimization using Jensen׳ s model. Renewable and Sustainable Energy Reviews.

[51] Breiman L (2001). Random Forests. Machine Learning.

[52] Li, L. *et al*. Crop yield forecasting and associated optimum lead time analysis based on multi-source environmental data across China. *Agricultural and Forest Meteorology* 308–309, 10.1016/j.agrformet.2021.108558 (2021).

[53] Wang LA, Zhou X, Zhu X, Dong Z, Guo W (2016). Estimation of biomass in wheat using random forest regression algorithm and remote sensing data. The Crop Journal.

[54] Feng P (2020). Dynamic wheat yield forecasts are improved by a hybrid approach using a biophysical model and machine learning technique. Agricultural and Forest Meteorology.

[55] Lu F, Sun Y, Hou F (2020). Using UAV Visible Images to Estimate the Soil Moisture of Steppe. Water.

[56] Wang S (2019). High spatial resolution monitoring land surface energy, water and CO2 fluxes from an Unmanned Aerial System. Remote Sensing of Environment.

[57] Chen Y (2014). Comparison of satellite-based evapotranspiration models over terrestrial ecosystems in China. Remote Sensing of Environment.

[58] Peralta N, Assefa Y, Du J, Barden C, Ciampitti I (2016). Mid-Season High-Resolution Satellite Imagery for Forecasting Site-Specific Corn Yield. Remote Sensing.

[59] Russello, H. Convolutional neural networks for crop yield prediction using satellite images. *IBM Center for Advanced Studies* (2018).

[60] You, J., Li, X., Low, M., Lobell, D. & Ermon, S. in *Proceedings of the AAAI Conference on Artificial Intelligence*.

[61] Moran PA (1950). Notes on continuous stochastic phenomena. Biometrika.

[62] Imran M, Stein A, Zurita-Milla R (2015). Using geographically weighted regression kriging for crop yield mapping in West Africa. International Journal of Geographical Information Systems.

[63] Harries K (2006). Extreme spatial variations in crime density in Baltimore County, MD. Geoforum.

[64] Ghulam A (2015). Remote Sensing Based Spatial Statistics to Document Tropical Rainforest Transition Pathways. Remote Sensing.

[65] Maimaitijiang M, Ghulam A, Sandoval JSO, Maimaitiyiming M (2015). Drivers of land cover and land use changes in St. Louis metropolitan area over the past 40 years characterized by remote sensing and census population data. International Journal of Applied Earth Observation Geoinformation.

[66] Cheng M (2021). Zenodo.

[67] Martens, B., Miralles, D. G., Lievens, H., Schalie, R. D. & Verhoest, N. GLEAM v3: Satellite-based land evaporation and root-zone soil moisture. *Geoscientific Model Development* **10**, 10.5194/gmd-10-1903-2017 (2017).

[68] Wang W, Cui W, Wang X, Chen X (2016). Evaluation of GLDAS-1 and GLDAS-2 forcing data and Noah model simulations over China at the monthly scale. Journal of Hydrometeorology.

[69] Chen X (2014). Development of a 10-year (2001–2010) 0.1° data set of land-surface energy balance for mainland China. Atmospheric Chemistry and Physics.

[70] Ramoelo, A. *et al*. Validation of Global Evapotranspiration Product (MOD16) using Flux Tower Data in the African Savanna, South Africa. *Remote Sensing* **6**, 10.3390/rs6087406 (2014).

[71] Yang X, Yong B, Ren L, Zhang Y, Long D (2017). Multi-scale validation of GLEAM evapotranspiration products over China via ChinaFLUX ET measurements. International Journal of Remote Sensing.

[72] Hu G, Jia L, Menenti M (2015). Comparison of MOD16 and LSA-SAF MSG evapotranspiration products over Europe for 2011. Remote Sensing of Environment.

[73] Khan MS, Liaqat UW, Baik J, Choi M (2018). Stand-alone uncertainty characterization of GLEAM, GLDAS and MOD16 evapotranspiration products using an extended triple collocation approach. Agricultural and Forest Meteorology.

[74] Glenn EP (2008). Scaling sap flux measurements of grazed and ungrazed shrub communities with fine and coarse-resolution remote sensing. Ecohydrology.

[75] Gamon JA (2015). Reviews and Syntheses: optical sampling of the flux tower footprint. Biogeosciences.

[76] Cai Y (2019). Integrating satellite and climate data to predict wheat yield in Australia using machine learning approaches. Agricultural and Forest Meteorology.

[77] Chen, X. *et al*. Prediction of Maize Yield at the City Level in China Using Multi-Source Data. *Remote Sensing* **13**, 10.3390/rs13010146 (2021).

[78] Guo Y (2021). Integrated phenology and climate in rice yields prediction using machine learning methods. Ecological Indicators.

[79] Yuan W (2016). Estimating crop yield using a satellite-based light use efficiency model. Ecological Indicators.

[80] Anandhi A (2016). Growing degree days – Ecosystem indicator for changing diurnal temperatures and their impact on corn growth stages in Kansas. Ecological Indicators.

[81] Wart JV (2013). Estimating Crop Yield Potential At National Scales. Field Crops Research.

[82] Kang, Y. S. *et al*. Yield prediction and validation of onion (Allium cepa L.) using key variables in narrowband hyperspectral imagery and effective accumulated temperature. *Computers and Electronics in Agriculture* **178**, 10.1016/j.compag.2020.105667 (2020).

[83] Long, D., Singh, V. P. & Li, Z.-L. How sensitive is SEBAL to changes in input variables, domain size and satellite sensor? *Journal of Geophysical Research: Atmospheres* **116**, 10.1029/2011jd016542 (2011).

[84] Liu Z, Wang L, Wang S (2014). Comparison of Different GPP Models in China Using MODIS Image and ChinaFLUX Data. Remote Sensing.

[85] Edreira J, Guilpart N, Sadras V, Cassman KG, Grassini P (2018). Water productivity of rainfed maize and wheat: A local to global perspective. Agricultural and Forest Meteorology.

[86] Li H (2021). Water Use Characteristics of Maize-Green Manure Intercropping Under Different Nitrogen Application Levels in the Oasis Irrigation Area Scientia Agricultura Sinica.

[87] Wang S, Ibrom A, Bauer-Gottwein P, Garcia M (2018). Incorporating diffuse radiation into a light use efficiency and evapotranspiration model: An 11-year study in a high latitude deciduous forest. Agricultural and Forest Meteorology.

[88] Cheng M (2022). Zenodo.

